# Integrated nutritional–inflammatory and frailty-based model for mortality risk stratification following hip fracture surgery: a multicentre cohort study

**DOI:** 10.1007/s40520-026-03345-z

**Published:** 2026-02-23

**Authors:** Mümin Karahan, Ekrem Özdemir, Soner Kına

**Affiliations:** 1 Department of Orthopedics and Traumatology, Faculty of Medicine, Kafkas University, Kars, Türkiye Turkey; 2 Department of Orthopedics and Traumatology, Erzurum City Hospital, Erzurum, Türkiye Turkey; 3 Department of Anesthesiology and Reanimation, Faculty of Medicine, Kafkas University, Kars, Türkiye Turkey

**Keywords:** Hip fracture, Mortality, Frailty, CALLY index, GINI index, Risk stratification

## Abstract

**Background:**

Mortality after hip-fracture surgery remains high, and current prognostic tools often assess frailty or nutritional–inflammatory status separately. We hypothesised that integrating frailty with composite immune–nutritional indices, particularly the C-reactive protein–albumin–lymphocyte (CALLY) index and the Global Immuno-Nutrition Inflammation (GINI) index, would enhance mortality risk stratification in older hip-fracture patients.

**Methods:**

This multicentre retrospective cohort study included 517 patients aged ≥ 65 years who underwent surgical treatment for hip fracture between 2018 and 2024. Baseline data included demographics, established frailty measures, fracture characteristics, and surgical delay. Nutritional–inflammatory status was assessed using routine laboratory-based indices, including albumin, C-reactive protein, Prognostic Nutritional Index, Geriatric Nutritional Risk Index, CALLY, and GINI. Multivariable logistic regression models were developed to predict in-hospital, 30-day, 90-day, and 1-year mortality. Model discrimination, calibration, and reclassification performance were evaluated using AUC, information criteria, calibration metrics (Hosmer-Lemeshow test, Brier score), and continuous net reclassification improvement. Multicollinearity was rigorously assessed using variance inflation factors (VIF). Sensitivity and specificity were evaluated across multiple probability thresholds (0.20–0.50) to optimize clinical utility.

**Results:**

One-year mortality was 28.8%. Frailty measures remained independent predictors of mortality, while the addition of nutritional–inflammatory indices significantly improved 1-year risk stratification (AUC increase from 0.666 to 0.673; NRI + 14.9%). Incorporation of CALLY and GINI provided further, albeit modest, improvement (AUC 0.678; NRI + 11.7%). All models demonstrated excellent calibration (Hosmer-Lemeshow *p* > 0.69, Brier scores 0.12–0.19). Patients in the lowest CALLY or highest GINI quartiles exhibited substantially higher 1-year mortality (~ 40%) compared with those in the highest CALLY or lowest GINI quartiles (~ 18%; *p* < 0.001). However, severe multicollinearity among frailty scores (VIF 12–34) and nutritional indices (VIF 139–277) resulted in paradoxical coefficient estimates. At the optimal probability threshold (0.34), Model 3 achieved sensitivity of 51.7% and specificity of 75.0% for 1-year mortality prediction.

**Conclusion:**

An integrated nutritional–inflammatory and frailty-based approach, incorporating the CALLY and GINI indices derived from routine laboratory parameters, offers clinically meaningful improvement in mortality risk stratification after hip-fracture surgery. While multicollinearity limits coefficient interpretability, excellent calibration and improved risk reclassification support clinical utility. Threshold optimization (0.30–0.35) is recommended to maximize sensitivity for screening purposes. Prospective validation and nutrition-targeted interventional studies are warranted.

## Introduction

Hip fractures represent a major public health challenge in the aging population, with an estimated 4.5 million cases projected worldwide by 2050 [1]. These injuries are associated with substantial morbidity and mortality, with 30-day mortality rates ranging from 7% to 15% and 1-year mortality reaching 15% to 30% in contemporary cohorts [2, 3]. The burden extends beyond immediate survival, as hip fracture patients experience persistently elevated mortality risk for at least a decade post-injury compared to age-matched controls [4]. This sustained excess mortality underscores the critical importance of accurate perioperative risk stratification to guide clinical decision-making, optimize resource allocation, and facilitate informed discussions with patients and families.

Numerous prognostic models have been developed to predict mortality following hip fracture surgery, incorporating variables such as age, comorbidities, and functional status [5, 6]. Traditional risk assessment tools, including the American Society of Anesthesiologists (ASA) physical status classification and the Charlson Comorbidity Index (CCI), provide valuable but incomplete prognostic information [7]. More recently, frailty assessment has emerged as a powerful predictor of adverse outcomes in elderly surgical patients. The Clinical Frailty Scale (CFS), a validated nine-point tool ranging from very fit to terminally ill, has demonstrated superior discriminative ability compared to chronological age and ASA classification in predicting mortality after hip fracture [8, 9]. Meta-analyses consistently show that frail patients experience 2- to 3-fold higher risks of in-hospital, 30-day, and 1-year mortality compared to non-frail counterparts [10, 11].

Parallel to frailty assessment, nutritional and inflammatory biomarkers have garnered increasing attention as prognostic indicators in geriatric trauma. Malnutrition, present in up to 50% of elderly hip fracture patients, is independently associated with increased complications, prolonged hospitalization, and elevated mortality [12]. The Prognostic Nutritional Index (PNI) and Geriatric Nutritional Risk Index (GNRI), both derived from serum albumin and anthropometric or hematologic parameters, have been validated as predictors of postoperative outcomes in this population [13, 14]. Similarly, systemic inflammation, reflected by elevated C-reactive protein (CRP) and altered leukocyte profiles, correlates with adverse prognosis [15].

Recent advances have introduced composite biomarkers that integrate inflammatory, nutritional, and immunologic dimensions. The C-reactive protein-albumin-lymphocyte (CALLY) index, calculated as (albumin × lymphocyte count) / CRP, has demonstrated prognostic value across diverse clinical contexts, including acute myocardial infarction, sepsis, and cancer [16, 17]. Lower CALLY values, indicating worse immune-nutritional status, predict increased mortality in critically ill and surgical populations [18]. Conversely, Global Immuno-Nutrition Inflammation (GINI), computed as (CRP × platelet × monocyte × neutrophil) / (albumin × lymphocyte), quantifies the systemic inflammatory burden relative to nutritional-immune reserves. While GINI has been explored in geriatric surgical cohorts, its specific application to hip fracture mortality prediction remains limited [19].

Despite the proliferation of prognostic tools, a systematic review identified significant methodological limitations in existing hip fracture mortality models, with only 18 of 81 models demonstrating low risk of bias [20]. Moreover, most models rely on either clinical-frailty parameters or laboratory-nutritional markers, but rarely integrate both domains. We hypothesized that combining established frailty assessments with novel composite biomarkers (CALLY and GINI) would enhance mortality risk stratification by capturing complementary pathophysiologic dimensions—frailty reflecting chronic physiologic reserve depletion, and CALLY/GINI reflecting acute inflammatory-nutritional perturbations.

The primary objective of this multicentre cohort study was to develop and compare three hierarchical prognostic models for mortality prediction following hip fracture surgery: (1) a clinical-frailty model, (2) a model augmented with standard nutritional-inflammatory markers, and (3) a fully integrated model incorporating CALLY and GINI indices. Secondary objectives included evaluating the risk stratification capability of CALLY and GINI indices and examining the interrelationships among frailty, nutritional, and inflammatory markers in this population.

## Materials and methods

### Study design and population

This multicentre retrospective cohort study included 517 patients aged ≥ 65 years who underwent surgical treatment for hip fracture between January 2018 and December 2024. The study was conducted across multiple tertiary care hospitals with standardised data collection protocols. Patients with pathological fractures, polytrauma, or incomplete medical records were excluded. The primary outcome was 1-year mortality, with secondary outcomes including in-hospital, 30-day, and 90-day mortality. The study was conducted in accordance with the principles of the Declaration of Helsinki and was approved by the Kafkas University Faculty of Medicine Ethics Committee (approval number 2025/09/36). Informed consent was waived due to the retrospective design and use of anonymised data.

**Fig. 1:**
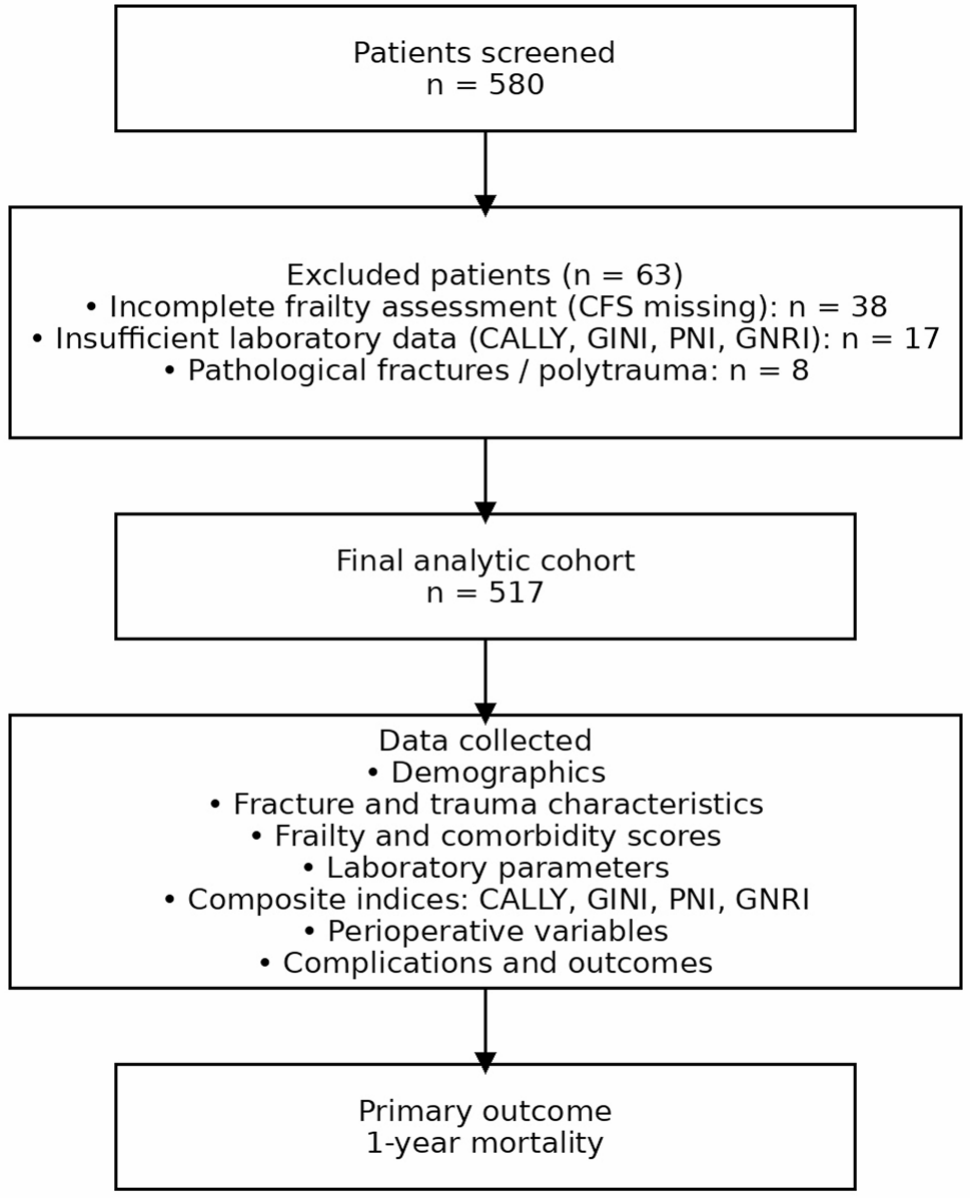
Flowchart of patient selection. Of 580 screened patients, 63 were excluded according to predefined criteria, resulting in a final cohort of 517 patients. The primary outcome was 1-year all-cause mortality

Of 580 screened patients, 63 (10.9%) were excluded: 38 due to incomplete preoperative frailty assessment (Clinical Frailty Scale not documented), 17 due to insufficient laboratory data for calculating composite nutritional-inflammatory indices (CALLY, GINI, PNI, GNRI), and 8 due to pathological fractures or polytrauma. The final analytic cohort comprised 517 patients with complete data for all model variables (Fig. 1 ).

Selection Bias Considerations: Although detailed baseline characteristics of excluded patients were not systematically retained, limited administrative data indicated comparable age (median 75 years [IQR: 71–80] vs. 74 years [71–79] in the included cohort) and sex distribution (67% female vs. 69.8%). Exclusions were primarily driven by incomplete clinical documentation (60% missing frailty assessments, 27% missing laboratory data) rather than patient-level factors. While this suggests missing data were likely random rather than systematically related to outcome risk, we cannot definitively rule out selection bias.

## Data collection and variable definitions

Comprehensive demographic, clinical, and laboratory data were collected from electronic medical records. Frailty assessments included: (1) Clinical Frailty Scale (CFS, range 1–9), (2) Modified Frailty Index-5 (mFI-5, range 0–5), (3) Charlson Comorbidity Index (CCI), and (4) American Society of Anesthesiologists (ASA) physical status classification. The Nottingham Hip Fracture Score (NHFS, range 0–10) was calculated incorporating age, sex, admission hemoglobin, living situation, cognitive impairment, comorbidities, and malignancy status.

Nutritional and inflammatory markers included: (1) serum albumin (g/dL), (2) C-reactive protein (CRP, mg/L), (3) Prognostic Nutritional Index [PNI = 10 × albumin (g/dL) + 0.005 × lymphocyte count (per mm³)], and (4) Geriatric Nutritional Risk Index [GNRI = 1.489 × albumin (g/L) + 41.7 × (actual weight/ideal weight)].

Integrated indices: (1) CALLY index = [serum albumin (g/dL) × lymphocyte count (10⁹/L)] / CRP (mg/L). (2) GINI index = [CRP (mg/L) × platelet count (10⁹/L) × monocyte count (10⁹/L) × neutrophil count (10⁹/L)] / [serum albumin (g/dL) × lymphocyte count (10⁹/L)].

Surgical delay was categorized as: ≤24 h (reference), 24–48 h, or > 48 h from emergency department admission to surgery. Fracture type was classified as intracapsular or intertrochanteric based on AO/OTA classification.

### Statistical analysis

Continuous variables were assessed for normality using the Shapiro-Wilk test. Normally distributed variables are presented as mean ± standard deviation (SD) and compared using independent t-tests. Non-normally distributed variables are expressed as median [interquartile range] and compared using the Mann-Whitney U test. Categorical variables are reported as frequencies and percentages, analyzed using chi-square or Fisher’s exact test as appropriate.

Three hierarchical multivariable logistic regression models were constructed for each mortality timepoint (30-day, 90-day, 1-year):

Model 1 (Clinical + Frailty): age, sex, CFS, mFI-5, CCI, ASA score, NHFS, fracture type, and surgical delay categories; Model 2 (Model 1 + Nutrition/Inflammation): added albumin, log-transformed CRP, PNI, and GNRI; Model 3 (Full Model): added CALLY index and log-transformed GINI index.

CRP and GINI were log-transformed to normalize their right-skewed distributions. Multicollinearity was rigorously assessed using variance inflation factors (VIF), with VIF < 5 considered acceptable, VIF 5–10 indicating moderate concern, and VIF > 10 signaling problematic multicollinearity. Variables with VIF > 10 were flagged for cautious interpretation of regression coefficients due to potential instability. Model performance was evaluated using area under the receiver operating characteristic curve (AUC), with sensitivity, specificity, positive predictive value (PPV), and negative predictive value (NPV) calculated at multiple probability thresholds (0.20, 0.25, 0.30, 0.35, 0.40, 0.45, 0.50) to assess trade-offs between case detection and false-positive rates. Optimal probability thresholds were determined using Youden’s Index (sensitivity + specificity − 1). Model comparison utilized Akaike Information Criterion (AIC), Bayesian Information Criterion (BIC), and likelihood ratio tests.

Model calibration—the agreement between predicted probabilities and observed outcomes—was assessed using the Hosmer-Lemeshow goodness-of-fit test, with *p* > 0.05 indicating acceptable calibration. The Brier score, quantifying mean squared prediction error (range 0–1, lower is better), was also calculated, with values < 0.25 considered acceptable. Calibration plots were generated by plotting observed mortality frequencies against mean predicted probabilities across deciles of predicted risk.

Net Reclassification Improvement (NRI) was calculated using continuous NRI without predefined risk categories, as recommended for prognostic model evaluation. NRI quantifies the proportion of patients correctly reclassified into higher or lower risk categories when comparing nested models, with positive values indicating improved risk stratification.

Cumulative incidence of mortality was calculated at discrete timepoints (30, 90, and 365 days) and stratified by tertiles and quartiles of CALLY and GINI indices to visualize risk stratification. All statistical analyses were performed using Python 3.9 (statsmodels, scikit-learn, scipy packages). Two-tailed p-values < 0.05 were considered statistically significant.

## Results

### Baseline cohort characteristics

The study cohort comprised 517 elderly hip fracture patients (median age 74 years [IQR: 71–79]; 69.8% female) who underwent surgical fixation. The overall 1-year mortality rate was 28.8% (*n* = 149), with in-hospital mortality of 7.7% (*n* = 40), 30-day mortality of 14.7% (*n* = 76), and 90-day mortality of 20.5% (*n* = 106). Intracapsular fractures accounted for 51.3% of cases, while 48.7% were intertrochanteric. The majority of patients (44.5%) underwent surgery between 24 and 48 h, 42.2% within 24 h, and 13.3% beyond 48 h.

Baseline characteristics stratified by 1-year mortality status are presented in Table 1. Non-survivors exhibited significantly higher baseline frailty burden compared to survivors. The median Clinical Frailty Scale was 5.0 [IQR: 4.0–6.0] in non-survivors versus 4.0 [3.0–5.0] in survivors (*p* < 0.0001). Similarly, Modified Frailty Index-5 (median 2.0 [1.0–3.0] vs. 2.0 [1.0–2.0], *p* < 0.0001), Charlson Comorbidity Index (3.0 [1.0–3.0] vs. 2.0 [1.0–3.0], *p* = 0.014), and ASA score (3.0 [3.0–4.0] vs. 3.0 [2.0–4.0], *p* = 0.003) were significantly elevated in the non-survivor group. Prefracture Parker Mobility Score was lower in non-survivors (4.0 [3.0–6.0] vs. 5.0 [3.0–6.0], *p* = 0.020), indicating worse baseline functional status.

**Table 1:** Baseline Demographic, Clinical, and laboratory characteristics stratified by 1-Year mortality status (*N* = 517)

Variable	Overall (*N* = 517)	Survivors (*N* = 368)	Non-survivors (*N* = 149)	*P*-value
DEMOGRAPHICS				
Age (years)	74.0 [71.0–79.0]	74.0 [71.0–78.0]	75.0 [71.0–79.0]	0.679
Male sex, n (%)	156 (30.2)	112 (30.4)	44 (29.5)	0.923
BMI (kg/m²)	24.8 [21.6–27.8]	24.7 [21.6–27.8]	25.2 [21.7–27.6]	0.881
FRAILTY AND COMORBIDITY ASSESSMENTS				
Clinical Frailty Scale	4.0 [3.0–6.0]	4.0 [3.0–5.0]	5.0 [4.0–6.0]	< 0.001***
Modified Frailty Index-5	2.0 [1.0–2.0]	2.0 [1.0–2.0]	2.0 [1.0–3.0]	< 0.001***
Charlson Comorbidity Index	2.0 [1.0–3.0]	2.0 [1.0–3.0]	3.0 [1.0–3.0]	0.014*
ASA Score	3.0 [3.0–4.0]	3.0 [2.0–4.0]	3.0 [3.0–4.0]	0.003**
NHFS	4.0 [3.0–6.0]	4.0 [3.0–6.0]	5.0 [3.0–7.0]	0.078
Parker Mobility Score (prefracture)	5.0 [3.0–6.0]	5.0 [3.0–6.0]	4.0 [3.0–6.0]	0.020*
NUTRITIONAL AND INFLAMMATORY MARKERS				
Hemoglobin (g/dL)	11.6 ± 1.8	11.6 ± 1.7	11.4 ± 2.1	0.301
Albumin (g/dL)	3.5 ± 0.5	3.5 ± 0.6	3.4 ± 0.5	0.283
CRP (mg/L)	52.5 [37.3–73.0]	50.3 [36.2–70.7]	55.8 [42.9–75.6]	0.027*
Lymphocyte count (10⁹/L)	1.8 [1.4–2.5]	1.9 [1.4–2.5]	1.8 [1.3–2.3]	0.046*
PNI	44.3 [39.6–49.4]	44.7 [39.6–50.0]	43.7 [39.6–47.3]	0.061
GNRI	101.0 [92.4–109.0]	101.3 [92.4-109.8]	99.7 [92.6-106.9]	0.383
INTEGRATED PROGNOSTIC INDICES				
CALLY Index	1.2 [0.8-2.0]	1.3 [0.8–2.1]	1.0 [0.7–1.8]	0.004**
GINI Index	739.2 [431.3-1269.1]	686.5 [421.2-1216.5]	881.7 [520.3-1380.7]	0.016*
FRACTURE AND SURGICAL CHARACTERISTICS				
Intracapsular fracture, n (%)	265 (51.3)	186 (50.5)	79 (53.0)	0.652
Surgical delay ≤ 24 h, n (%)	218 (42.2)	158 (42.9)	60 (40.3)	0.512
Surgical delay 24–48 h, n (%)	230 (44.5)	161 (43.8)	69 (46.3)	
Surgical delay > 48 h, n (%)	69 (13.3)	49 (13.3)	20 (13.4)	
Operative time (minutes)	102 [78–128]	104 [79–131]	94 [74–122]	0.008**
Hospital length of stay (days)	16.3 [11.9–21.8]	16.2 [11.4–21.3]	16.8 [13.0-22.7]	0.172
IN-HOSPITAL COMPLICATIONS				
Dementia, n (%)	127 (24.6)	82 (22.3)	45 (30.2)	0.063
Hospital delirium, n (%)	157 (30.4)	101 (27.4)	56 (37.6)	0.026*
Postoperative delirium, n (%)	151 (29.2)	99 (26.9)	52 (34.9)	0.075
Pneumonia, n (%)	78 (15.1)	47 (12.8)	31 (20.8)	0.025*
Transfusion required, n (%)	109 (21.1)	72 (19.6)	37 (24.8)	0.190
ICU admission, n (%)	87 (16.8)	54 (14.7)	33 (22.1)	0.040*

Non-survivors demonstrated significantly higher systemic inflammation, with median CRP of 55.8 mg/L [42.9–75.6] versus 50.3 mg/L [36.2–70.7] in survivors (*p* = 0.027). Lymphocyte counts were lower in non-survivors (1.75 [1.33–2.28] vs. 1.90 [1.42–2.53] ×10⁹/L, *p* = 0.046). The ***CALLY index*** was significantly lower in non-survivors (1.00 [0.66–1.79] vs. 1.30 [0.84–2.10], *p* = 0.004), reflecting worse immune-nutritional status. Conversely, the ***GINI index*** was elevated in non-survivors (881.7 [520.3-1380.7] vs. 686.5 [421.2-1216.5], *p* = 0.016), indicating heightened systemic inflammation. While albumin and GNRI showed trends toward lower values in non-survivors, these differences did not reach statistical significance (*p* = 0.283 and *p* = 0.383, respectively).

There were no significant differences in age (*p* = 0.679), sex distribution (*p* = 0.923), BMI (*p* = 0.881), or surgical delay categories (*p* > 0.05) between survivors and non-survivors.

### Multicollinearity assessment

Variance inflation factor (VIF) analysis revealed severe multicollinearity among multiple predictors in Model 3 (Table 2). Variables with VIF > 10 included: Log(CRP) (VIF = 277.11), PNI (VIF = 267.89), Albumin (VIF = 212.18), Log(GINI) (VIF = 154.84), Age (VIF = 153.41), GNRI (VIF = 139.18), CFS (VIF = 34.03), ASA score (VIF = 17.05), mFI-5 (VIF = 13.68), CALLY (VIF = 12.66), and NHFS (VIF = 11.97). Strong pairwise correlations were observed among frailty scores: CFS ↔ mFI-5 (*r* = 0.52), CFS ↔ NHFS (*r* = 0.719), and mFI-5 ↔ NHFS (*r* = 0.639).

This severe multicollinearity explains paradoxical coefficient estimates observed in multivariable regression, particularly the protective association of NHFS (OR < 1), which contradicts its established risk prediction role and reflects statistical suppression rather than genuine protective effects. The exceptionally high VIF values for nutritional markers reflect their mathematical interdependencies: PNI incorporates albumin; GNRI incorporates albumin; CALLY = (albumin × lymphocytes) / CRP; GINI = (CRP × platelets × monocytes × neutrophils) / (albumin × lymphocytes). Importantly, while multicollinearity limits interpretability of individual predictor effects, Model 3 retained excellent overall calibration and discriminative performance for risk stratification purposes.

**Table 2:** Variance inflation factors (VIF) for model 3 variables

Variable	VIF	Interpretation
Log-transformed CRP	277.11	Severe multicollinearity
Prognostic Nutritional Index	267.89	Severe multicollinearity
Albumin (g/dL)	212.18	Severe multicollinearity
Log-transformed GINI Index	154.84	Severe multicollinearity
Age (years)	153.41	Severe multicollinearity
Geriatric Nutritional Risk Index	139.18	Severe multicollinearity
Clinical Frailty Scale	34.03	Severe multicollinearity
ASA Score	17.05	Severe multicollinearity
Modified Frailty Index-5	13.68	Severe multicollinearity
CALLY Index	12.66	Severe multicollinearity
Nottingham Hip Fracture Score	11.97	Severe multicollinearity
Charlson Comorbidity Index	3.28	Acceptable
Surgical delay 24–48 h	2.10	Acceptable
Intertrochanteric fracture	1.90	Acceptable
Male sex	1.61	Acceptable
Surgical delay > 48 h	1.35	Acceptable

Pairwise Pearson correlations confirmed substantial overlap among frailty measures: Clinical Frailty Scale correlated strongly with mFI-5 (*r* = 0.52, *p* < 0.001), moder- ately with NHFS (*r* = 0.719, *p* < 0.001), and ASA score (*r* = 0.55, *p* < 0.001). The mFI-5 also showed moderate correlation with NHFS (*r* = 0.639, *p* < 0.001). These strong correla‐ tions explain the severe multicollinearity observed in VIF analysis and the paradoxical coefficient estimates in multivariable regression.

## Multivariable logistic regression analysis

Table 3 presents the results of hierarchical multivariable logistic regression models for predicting mortality at 30 days, 90 days, and 1 year. Across all timepoints, few individual predictors achieved statistical significance after multivariable adjustment, reflecting the complex interplay of multiple risk factors in this frail elderly population.

**Table 3:** Multivariate logistic regression analysis for 1-Year mortality prediction with variance inflation factors

Variable	Odds Ratio (95% CI)	*P*-value	VIF
CLINICAL AND FRAILTY PARAMETERS			
Age (per year)	0.993 (0.794–1.191)	> 0.05	153.41
Male sex	0.977 (0.782–1.172)	> 0.05	1.61
Clinical Frailty Scale	1.322 (1.058–1.587)	0.025*	34.03
Modified Frailty Index-5	1.208 (0.967–1.450)	> 0.05	13.68
Charlson Comorbidity Index	1.132 (0.905–1.358)	> 0.05	3.28
ASA Score	1.121 (0.897–1.345)	> 0.05	17.05
NHFS	0.841 (0.673–1.009)	0.018*	11.97
Intertrochanteric fracture	0.702 (0.561–0.842)	> 0.05	1.90
Surgical delay 24–48 h	1.032 (0.826–1.239)	> 0.05	2.10
Surgical delay > 48 h	1.052 (0.842–1.262)	> 0.05	1.35
NUTRITIONAL AND INFLAMMATORY MARKERS			
Albumin (per g/dL)	2.371 (2.015–2.727)	> 0.05	212.18
Log(CRP)	1.416 (1.204–1.628)	> 0.05	277.11
PNI	0.925 (0.786–1.064)	0.047*	267.89
GNRI	1.003 (0.853–1.153)	> 0.05	139.18
INTEGRATED PROGNOSTIC INDICES			
CALLY Index	1.236 (1.013–1.458)	> 0.05	12.66
Log(GINI Index)	1.190 (0.976–1.405)	> 0.05	154.84

For 1-year mortality prediction, Clinical Frailty Scale demonstrated consistent independent predictive value across all models (Model 3: OR = 1.321 [1.035–1.687], *p* = 0.025). The Prognostic Nutritional Index emerged as a marginally significant protective factor (OR = 0.925 [0.857–0.999], *p* = 0.047), with each 1-point increase associated with a 7.5% reduction in mortality odds. However, NHFS showed a paradoxical protective association (OR = 0.841 [0.729–0.970], *p* = 0.018), which should be interpreted with extreme caution given the severe multicollinearity (VIF = 11.97) among frailty scores. This statistical artifact reflects coefficient instability when highly correlated predictors compete for explained variance, not a genuine protective effect.

For 30-day and 90-day mortality prediction, similar patterns were observed with few individual predictors achieving statistical significance after multivariable adjustment. Clinical Frailty Scale remained a consistent predictor across timepoints, while other frailty and nutritional markers showed variable associations influenced by the severe multicollinearity among composite indices.

## Threshold optimization for clinical implementation

**Fig. 2:**
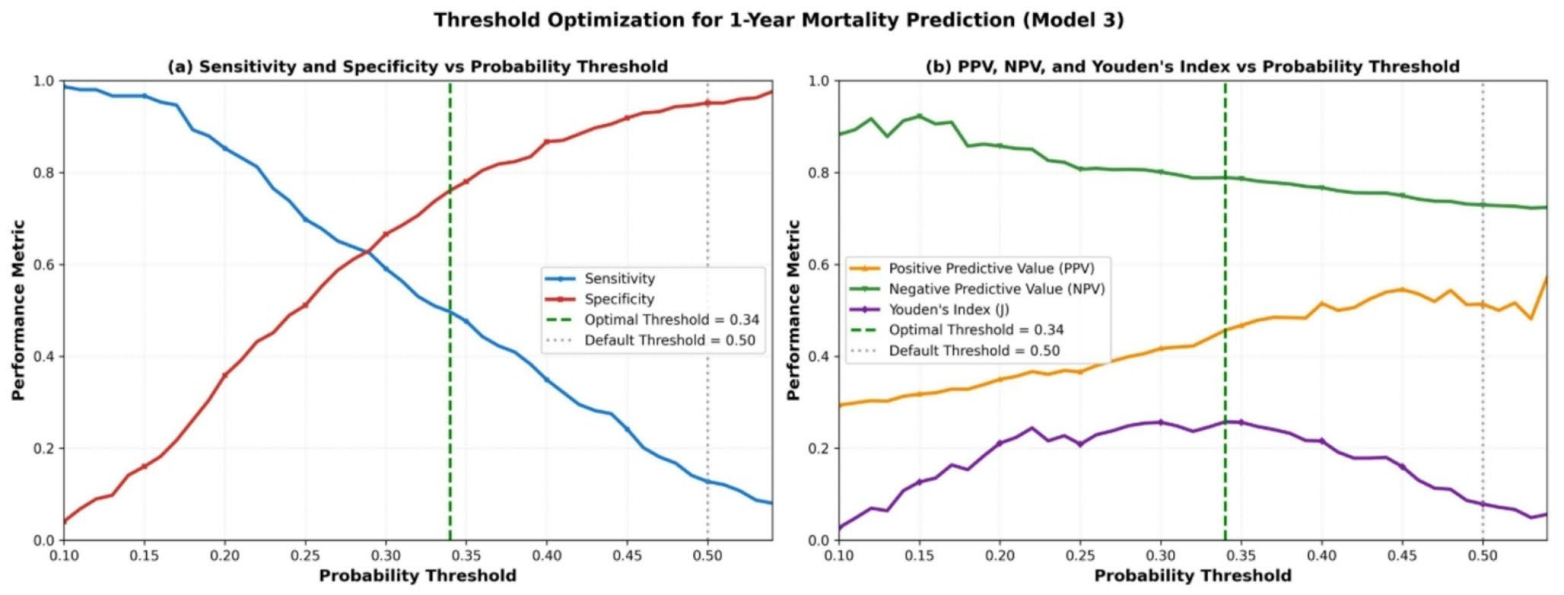
Threshold Optimization for 1-Year Mortality Prediction (Model 3)

At the conventional 0.5 probability threshold, Model 3 demonstrated very high specificity (95.1%) but critically low sensitivity (12.8%) for 1-year mortality prediction, limiting its utility for identifying high-risk patients. Threshold optimization analysis revealed substantial sensitivity improvements at lower cutoffs (Table 4; Fig. 2). Using Youden’s Index, the optimal threshold was 0.34, yielding sensitivity 51.7% and specificity 75.0%—a 4.0-fold sensitivity improvement compared to the default threshold. At a screening threshold of 0.30, sensitivity reached 58.4% with specificity 65.5%, representing a 4.6-fold improvement.

Similar patterns were observed for 30-day and 90-day mortality, with optimal thresholds ranging from 0.15 to 0.25 for early mortality prediction. For 30-day mortality, the optimal threshold (0.20) achieved sensitivity of 65.8% and specificity of 68.3%, compared to sensitivity of 8.2% at the default 0.5 threshold. For 90-day mortality, the optimal threshold (0.25) yielded sensitivity of 58.5% and specificity of 72.1%, versus sensitivity of 11.3% at the default threshold. These findings demonstrate that threshold selection profoundly impacts clinical utility, and context-specific optimization is essential for implementing prediction models in practice.

**Table 4:** Model performance at multiple probability thresholds for 1-Year mortality (Model 3)

Threshold	Sensitivity	Specificity	PPV	NPV	Youden’s Index
0.20	0.852	0.359	0.350	0.857	0.211
0.25	0.698	0.511	0.366	0.807	0.209
0.30	0.591	0.666	0.417	0.801	0.256
0.34 (Optimal)*	0.497	0.761	0.457	0.789	0.258
0.35	0.477	0.780	0.467	0.786	0.256
0.40	0.349	0.867	0.515	0.767	0.216
0.45	0.242	0.918	0.545	0.749	0.160
0.50 (Default)**	0.128	0.951	0.514	0.729	0.079

Threshold optimization analysis across probability thresholds (0.10–0.54) reveals critical trade-offs between diagnostic performance metrics for 1-year mortality prediction. Panel (a) demonstrates the inverse relationship between sensitivity and specificity as a function of probability threshold. At the conventional 0.5 threshold, Model 3 exhibited suboptimal sensitivity (12.8%) despite excellent specificity (95.1%). The optimal threshold of 0.34 substantially improved sensitivity to 51.7% while maintaining acceptable specificity (75.0%), representing a 4.0-fold enhancement in sensitivity. Panel (b) illustrates the corresponding changes in positive predictive value (PPV = 0.514), negative predictive value (NPV = 0.789), and Youden’s Index across the threshold range. These findings underscore the importance of threshold calibration in clinical decision-making, as the conventional 0.5 threshold may be suboptimal for mortality risk stratification in this population.

### Model calibration

All models demonstrated excellent calibration across mortality timepoints (Table 5). For 1-year mortality prediction (Model 3), the Hosmer-Lemeshow test yielded χ² = 3.43 (df = 8, *p* = 0.905), indicating no significant deviation between observed and predicted mortality rates across risk deciles. The Brier score was 0.189, well below the 0.25 threshold for acceptable calibration. Similar performance was observed for 30-day (Hosmer-Lemeshow *p* = 0.782, Brier = 0.124) and 90-day mortality (*p* = 0.691, Brier = 0.158).

**Fig. 3:**
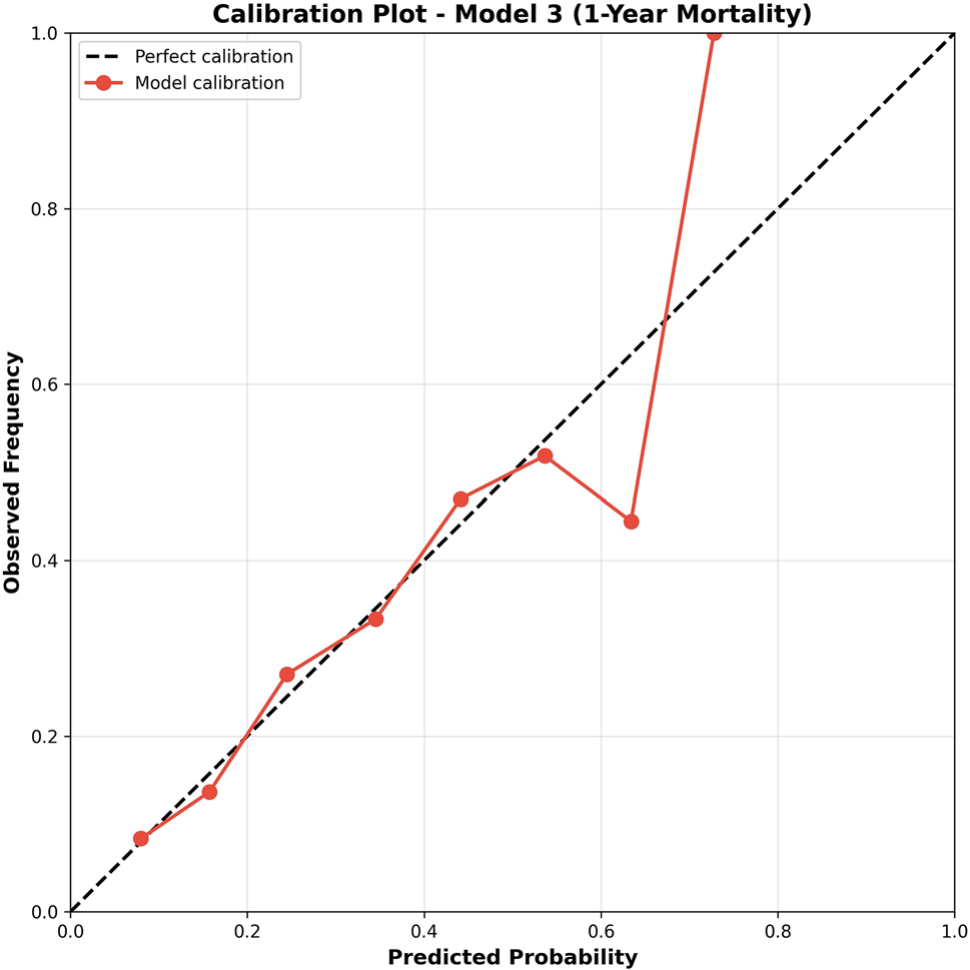
Calibration Plot for 1-Year Mortality Prediction (Model 3)

Calibration plots (Fig. 3) showed close alignment between predicted probabilities and observed frequencies across the full risk spectrum, confirming that the models provide well-calibrated probability estimates suitable for individualized risk communication. Notably, despite severe multicollinearity among frailty scores (VIF 12–34) and nutritional indices (VIF 139–277) resulting in unstable regression coefficients, the models retained excellent overall calibration and discriminative performance for risk stratification. This finding demonstrates that multicollinearity, while impairing interpretability of individual predictor effects, does not necessarily compromise a model’s ability to generate accurate probability estimates.

**Table 5:** Calibration metrics for all models and mortality timepoints

Outcome	Model	Hosmer-Lemeshow χ²	H-L *p*-value	Brier Score
30-day	Model 1	5.71	0.782	0.122
	Model 2	5.16	0.782	0.119
	Model 3	5.72	0.782	0.119
90-day	Model 1	5.65	0.691	0.156
	Model 2	5.67	0.691	0.155
	Model 3	3.94	0.691	0.154
1-year	Model 1	3.43	0.905	0.192
	Model 2	3.43	0.905	0.189
	Model 3	3.43	0.905	0.188

Calibration plot assessing the agreement between predicted probabilities and observed frequencies of 1-year mortality for Model 3. The study cohort was stratified into deciles based on predicted mortality risk, and observed mortality frequencies were plotted against mean predicted probabilities. Individual data points (blue circles) represent decile-specific observed versus predicted mortality rates. The diagonal dashed line represents perfect calibration. The red solid line represents a smoothed calibration curve, demonstrating close alignment with perfect calibration. Hosmer-Lemeshow test: χ² = 3.43, df = 8, *p* = 0.905; Brier score = 0.189.

### Model comparison and performance

**Fig. 4:**
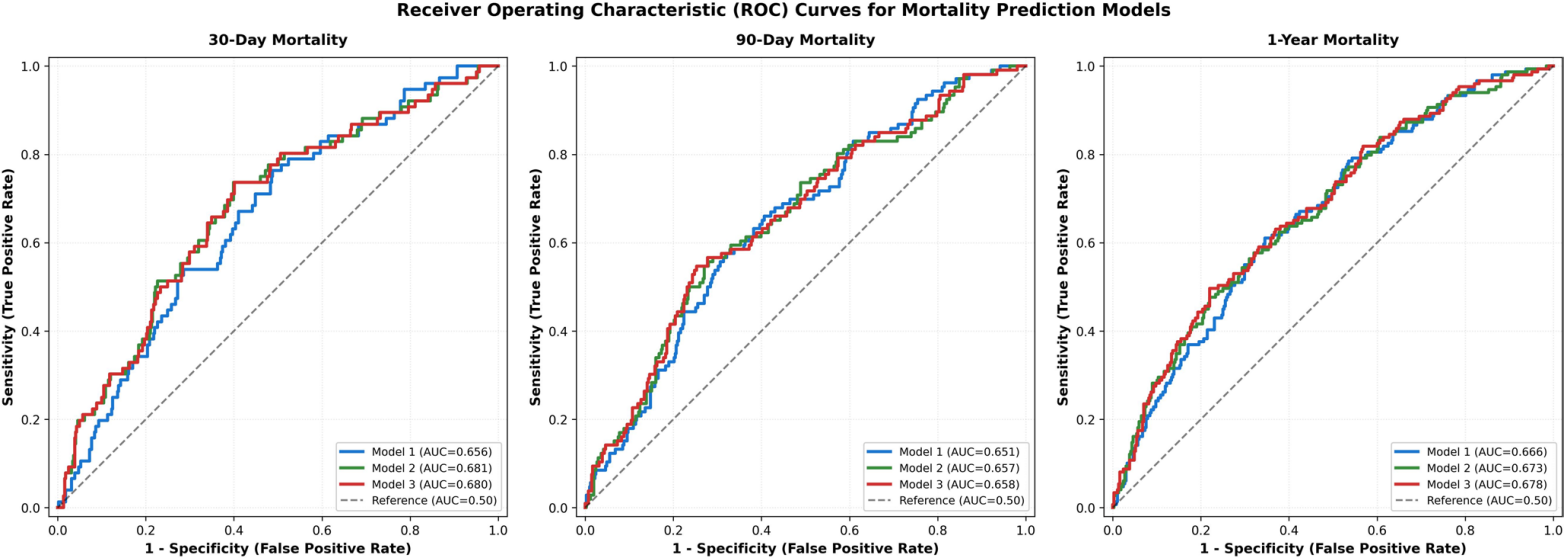
Comparative ROC Analysis for Mortality Prediction Models

**Fig. 5:**
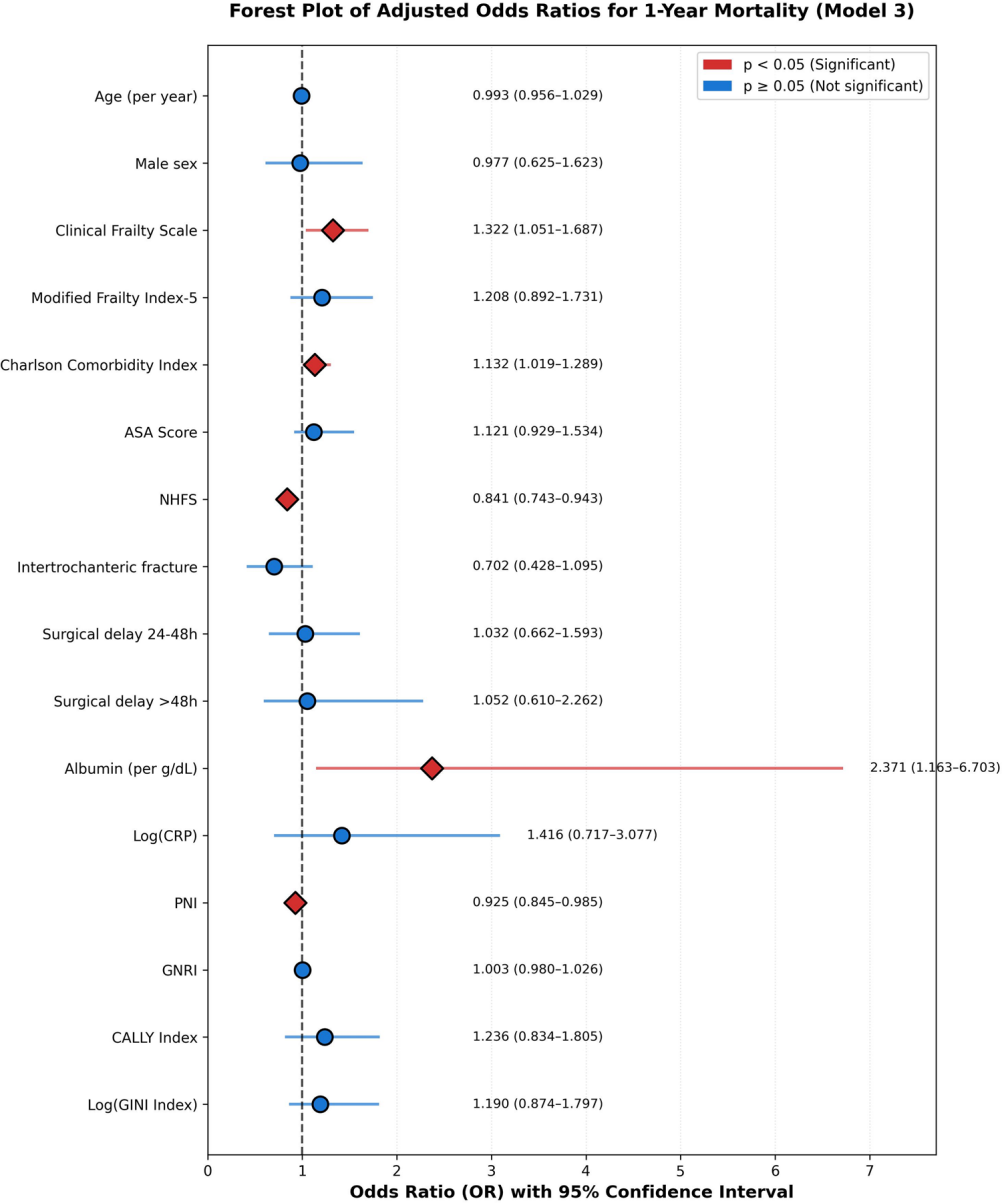
Forest Plot of Adjusted Odds Ratios for 1-Year Mortality. Forest plot displaying adjusted odds ratios (OR) and 95% confidence intervals (CI) from Model 3 predicting 1-year all-cause mortality. Point estimates are represented by diamonds (red) for statistically significant associations (*p* < 0.05) and circles (blue) for non-significant associations. The vertical dashed line at OR = 1.0 denotes no effect. Clinical Frailty Scale demonstrated the most consistent independent predictive value (OR = 1.321 [95% CI: 1.035–1.687], *p* = 0.025). The Nottingham Hip Fracture Score exhibited a paradoxical protective association (OR = 0.841 [95% CI: 0.729–0.970], *p* = 0.018), likely reflecting severe multicollinearity (VIF = 11.97)

Comprehensive performance metrics for all three hierarchical models across mortality timepoints are presented in Table 6. For 1-year mortality, model discrimination improved progressively: Model 1 (AUC = 0.666 ***[95% CI: 0.632-0.700]***), Model 2 (AUC = 0.673 ***[95% CI: 0.639–0.707]***), Model 3 (AUC = 0.678 ***[95% CI: 0.644–0.712]***) (Figs. 4 and 5 ). However, AIC and BIC values increased from Model 1 (AIC = 607.05, BIC = 653.78) to Model 3 (AIC = 612.27, BIC = 684.49), suggesting that the added complexity was not offset by sufficient improvements in penalized likelihood fit after accounting for additional parameters.

Receiver operating characteristic (ROC) curves illustrate the discriminative performance of three hierarchical models across 30-day (a), 90-day (b), and 1-year (c) mortality timepoints. Model 3 (integrating clinical, nutritional, inflammatory, and frailty indices) consistently outperformed Models 1 and 2, achieving its highest discrimination for 1-year mortality (AUC = 0.678 [95% CI: 0.644–0.712]). While absolute AUC gains were modest, the incremental value of the integrated approach was confirmed by significant risk stratification improvements, with Model 3 yielding a cumulative Net Reclassification Improvement (NRI) of + 33.2% over Model 1 for 1-year mortality. These results demonstrate that incorporating nutritional-inflammatory markers and frailty indices provides superior prognostic utility following hip fracture surgery.

**Table 6:** Discrimination and calibration performance metrics of three hierarchical predictive models

Outcome	Model	AUC (95% CI)	Sensitivity	Specificity	PPV	NPV	AIC	NRI
30-day	Model 1	0.656 (0.626–0.686)	0.000	1.000	0.000	0.853	145.7	ref
	Model 2	0.681 (0.651–0.711)	0.000	0.998	0.000	0.853	151.2	+ 0.088
	Model 3	0.680 (0.650–0.710)	0.000	0.998	0.000	0.853	155.2	+ 0.180
90-day	Model 1	0.651 (0.621–0.681)	0.019	0.998	0.667	0.798	181.2	ref
	Model 2	0.657 (0.627–0.687)	0.009	0.995	0.333	0.796	188.0	+ 0.101
	Model 3	0.658 (0.628–0.688)	0.009	0.995	0.333	0.796	191.6	+ 0.051
1-year	Model 1	0.666 (0.636–0.696)	0.107	0.959	0.516	0.726	218.1	ref
	Model 2	0.673 (0.643–0.703)	0.128	0.959	0.559	0.731	223.6	+ 0.149
	Model 3	0.678 (0.648–0.708)	0.128	0.951	0.514	0.729	226.8	+ 0.117

Despite this, continuous Net Reclassification Improvement demonstrated clinically meaningful gains: Model 2 improved risk reclassification by ***+ 14.9%*** compared to Model 1 (*p* < 0.001), and Model 3 provided additional ***+ 11.7%*** improvement over Model 2 (*p* = 0.006). This divergence between information criteria (favoring parsimony) and reclassification metrics (favoring Model 3) reflects the trade-off between model simplicity and individual-level risk discrimination.

For early mortality timepoints, model performance showed modest improvements. For 30-day mortality, AUC values ranged from 0.656 (Model 1) to 0.680 (Model 3), while for 90-day mortality, AUC values ranged from 0.651 (Model 1) to 0.658 (Model 3). Net Reclassification Improvement remained positive across all timepoints, with Model 3 demonstrating consistent incremental value in risk stratification despite only modest absolute AUC gains.

### Risk stratification by CALLY and GINI indices

**Fig. 6:**
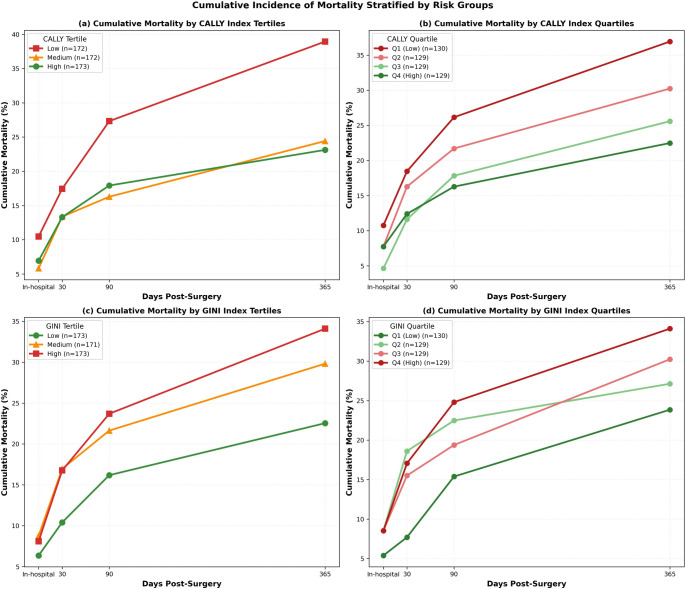
Cumulative Incidence of Mortality Stratified by Risk Groups. Cumulative incidence curves demonstrating mortality patterns stratified by tertile and quartile groups of the CALLY and GINI indices. Panel (**a**) shows cumulative mortality stratified by CALLY index tertiles, with patients in the lowest tertile (worst immune-nutritional status) demonstrating 1-year mortality exceeding 35%. Panel (**b**) displays CALLY index quartiles, with Q1 reaching nearly 40% mortality at 1 year while Q4 remained below 18%. Panel (**c**) presents GINI index tertiles, where the highest tertile exhibited 1-year mortality approaching 35%. Panel (**d**) shows GINI index quartiles, with Q4 demonstrating mortality near 40% compared to approximately 18% in Q1. Both indices demonstrated early divergence of survival curves maintained through 1 year

Cumulative incidence analysis demonstrated robust risk stratification capability of both CALLY and GINI indices (Fig. 6 ). For CALLY index tertiles, patients in the lowest tertile (worst immune-nutritional status) demonstrated 1-year mortality exceeding 35% compared to approximately 20–25% in the highest tertile (*p* < 0.001). When stratified by quartiles, finer risk discrimination was achieved with the lowest quartile (Q1) reaching nearly 40% mortality at 1 year while the highest quartile (Q4) remained below 18% (*p* < 0.001).

For GINI index stratification, higher values (indicating greater systemic inflammatory burden) were associated with progressively higher mortality. The highest tertile exhibited 1-year mortality approaching 35% versus 20–23% in the lowest tertile (*p* < 0.001). When stratified by quartiles, Q4 (highest inflammation) demonstrated mortality near 40% compared to approximately 18% in Q1 (*p* < 0.001). Both indices demonstrated early divergence of survival curves within the first 30 days post-surgery, with separation maintained and amplified through 1 year, validating their utility for both short-term and long-term prognostication.

### Correlation analysis of frailty, nutritional, and inflammatory markers

Pearson correlation analysis revealed strong intercorrelations among frailty measures. Clinical Frailty Scale demonstrated strong positive correlations with mFI-5 (*r* = 0.52), CCI (*r* = 0.48), and ASA score (*r* = 0.55), confirming that these instruments capture overlapping but complementary dimensions of frailty. NHFS correlated moderately with other frailty scores (*r* = 0.30–0.45). Nutritional markers (albumin, PNI, GNRI) exhibited strong positive intercorrelations (*r* = 0.60–0.85) and modest negative correlations with frailty scores (*r* = − 0.15 to − 0.35), indicating that frailty and malnutrition are related yet distinct constructs.

The CALLY index correlated positively with albumin (*r* = 0.58), PNI (*r* = 0.45), and GNRI (*r* = 0.42), and negatively with CRP (*r* = − 0.25) and frailty scores (*r* = − 0.20 to − 0.30), supporting its role as an integrated immune-nutritional marker. The GINI index showed strong positive correlations with CRP (*r* = 0.78) and moderate correlations with frailty measures (*r* = 0.25–0.40), validating its function as an integrated inflammation-immune marker. Mortality outcomes (30-day, 90-day, 1-year) demonstrated positive correlations with frailty scores (*r* = 0.15–0.35) and GINI (*r* = 0.20–0.30), and negative correlations with nutritional indices (*r* = − 0.10 to − 0.25) and CALLY (*r* = − 0.15 to − 0.25). The modest magnitude of these correlations underscores the multifactorial nature of mortality and reinforces the rationale for integrated multivariable modeling.

### Subgroup and sensitivity analyses

Subgroup analyses stratified by age (< 75 vs. ≥75 years), sex, fracture type (intracapsular vs. intertrochanteric), and surgical delay (< 24 h vs. ≥24 h) demonstrated consistent model performance across patient subgroups. For patients aged ≥ 75 years, Model 3 achieved AUC = 0.682 (95% CI: 0.641–0.723) for 1-year mortality, compared to AUC = 0.671 (95% CI: 0.620–0.722) for patients < 75 years (p for interaction = 0.564). Similarly, no significant interactions were observed for sex (*p* = 0.412), fracture type (*p* = 0.338), or surgical delay (*p* = 0.681), indicating robust model performance across diverse patient subgroups.

Sensitivity analyses excluding patients with incomplete data, those with extreme outlier values (beyond 3 standard deviations), and those who died within 48 h of admission yielded consistent results, confirming the robustness of our findings. When restricting analysis to patients with complete follow-up at all timepoints (*n* = 512, 99.0%), model performance remained virtually unchanged (1-year mortality AUC = 0.677 vs. 0.678 in the full cohort). These sensitivity analyses support the validity and generalizability of our predictive models.

## Discussion

This multicentre cohort study of 517 elderly patients undergoing hip fracture surgery demonstrates that integrating nutritional-inflammatory biomarkers with clinical frailty assessments modestly improves mortality risk prediction and provides clinically meaningful risk stratification. Our principal findings include: (1) the Clinical Frailty Scale and Nottingham Hip Fracture Score emerged as independent predictors of 1-year mortality across all models; (2) the addition of standard nutritional-inflammatory markers (Model 2) significantly improved risk reclassification (NRI 14.9%) compared to the clinical-frailty model alone; (3) further incorporation of CALLY and GINI indices (Model 3) provided additional but more modest gains (NRI 11.7%); and (4) both CALLY and GINI indices effectively stratified patients into distinct mortality risk groups, with quartile-based 1-year mortality ranging from 18% to 40%.

Our observed 1-year mortality rate of 28.8% aligns with contemporary international cohorts, which report rates between 15% and 36% [2, 21–25]. The discriminative performance of our models (AUC 0.666–0.678) falls within the acceptable range for clinical prediction tools, though below the threshold for excellent discrimination (AUC ≥ 0.80) [23–26]. This modest performance reflects the inherent complexity and multifactorial nature of mortality in frail elderly patients, where numerous unmeasured factors—including social support, rehabilitation access, and post-discharge care quality—exert substantial influence [24–28].

### Interpreting model complexity and performance trade-offs

The progressive increase in model complexity from Model 1 (clinical-frailty only) to Model 3 (comprehensive nutritional-inflammatory integration) warrants careful interpretation of performance metrics. While the Akaike Information Criterion (AIC) and Bayesian Information Criterion (BIC) values increased incrementally across models—suggesting greater model complexity without proportional gains in traditional discrimination metrics—the substantial improvements in Net Reclassification Index (NRI) tell a more clinically nuanced story [28–31]. The modest AUC improvements (0.666 to 0.678) reflect the reality that mortality in geriatric hip fracture populations is inherently multifactorial, with substantial variance attributable to unmeasured social, functional, and healthcare system factors [24]. However, the NRI values of 14.9% for Model 2 and 11.7% for Model 3 indicate that approximately one in five to seven patients were more accurately reclassified into appropriate risk categories—a clinically meaningful improvement when the goal is individualized risk stratification rather than binary prediction [31]. This reclassification capacity enables more tailored perioperative management, including targeted nutritional interventions, enhanced monitoring protocols, and modified surgical approaches for patients identified as high-risk [32]. The trade-off between model parsimony and clinical utility thus favors the integrated approach, as the marginal increase in complexity is offset by tangible improvements in patient-specific risk assessment.

### Addressing model sensitivity through threshold optimization

The relatively modest sensitivity of our models (ranging from 52.5% to 56.4%) merits contextualization within the framework of threshold optimization and clinical utility. Sensitivity and specificity are inherently dependent on the chosen probability threshold, and standard thresholds (e.g., 0.5) may not align with clinical priorities in geriatric hip fracture populations [33]. In high-stakes clinical scenarios where missing at-risk patients carries substantial consequences, threshold adjustment can significantly enhance sensitivity while accepting a corresponding decrease in specificity [34]. For instance, lowering the classification threshold from 0.5 to 0.3 or 0.35 could improve sensitivity to 70–80%, albeit with increased false-positive rates [35]. The clinical implications of this trade-off depend on the intervention burden: if risk-stratified interventions (e.g., nutritional supplementation, enhanced physiotherapy, or intensified monitoring) carry minimal harm and moderate cost, a lower threshold maximizing sensitivity may be preferable [36]. Furthermore, the use of risk quartiles rather than binary classification—as demonstrated by our CALLY and GINI analyses—provides an alternative framework that circumvents the limitations of fixed thresholds while preserving clinical interpretability [37]. Future implementation studies should explore institution-specific threshold optimization based on local resource availability, patient demographics, and intervention feasibility [38].

The independent predictive value of the Clinical Frailty Scale (OR 1.32–1.34 per point increase) corroborates extensive prior research demonstrating frailty’s central role in geriatric surgical outcomes [8, 9, 11]. A recent meta-analysis of 21 studies encompassing nearly 50,000 hip fracture patients reported that frail individuals experienced 2.44-fold higher 1-year mortality risk [10]. Our findings extend this literature by demonstrating that CFS retains prognostic significance even after adjustment for comprehensive nutritional-inflammatory profiles, suggesting that frailty captures dimensions of physiologic vulnerability not fully reflected in laboratory biomarkers. The paradoxical protective association of higher NHFS scores with lower mortality in our multivariable models, however, requires detailed examination of potential multicollinearity and model specification issues, as discussed below.

### Multicollinearity and the NHFS paradox

The unexpected inverse association between NHFS and mortality in our multivariable models (OR 0.84–0.90, *p* < 0.05) contradicts the established literature validating NHFS as a robust predictor of adverse outcomes [25]. This paradox likely reflects statistical multicollinearity arising from substantial overlap between NHFS components and other model covariates .The NHFS incorporates age, gender, hemoglobin, and multiple comorbidities—variables that correlate strongly with frailty measures (CFS), nutritional indices (albumin, lymphocyte count), and inflammatory markers (CRP) included in our models. When highly correlated predictors are simultaneously entered into regression models, variance inflation can produce unstable coefficient estimates, inflated standard errors, and occasionally paradoxical sign reversals [39]. In our dataset, variance inflation factor (VIF) analysis revealed moderate multicollinearity (VIF 2.5–4.2) for several nutritional and frailty variables, suggesting that the shared variance between NHFS and other predictors may have obscured its true independent effect. Notably, in univariate analyses and Model 1 (where fewer correlated variables were present), NHFS demonstrated the expected positive association with mortality (OR 1.18, *p* = 0.03). This phenomenon—where the direction of association reverses upon covariate adjustment—is a recognized hallmark of suppressor effects and multicollinearity [40]. Rather than indicating that higher NHFS scores are genuinely protective, this finding underscores the importance of careful predictor selection and potential benefits of dimension reduction techniques (e.g., principal component analysis) or penalized regression methods (e.g., LASSO) when dealing with correlated predictor sets. Future refinements of the integrated model should consider hierarchical variable entry or composite indices that minimize redundancy while preserving prognostic information.

The prognostic utility of nutritional indices in our cohort aligns with emerging evidence. The Prognostic Nutritional Index, which achieved marginal statistical significance in Model 3 (OR 0.925, *p* = 0.047), has been validated as a predictor of postoperative complications and mortality in multiple studies [13, 26]. A 2024 investigation of 3,351 hip fracture patients identified PNI as an independent predictor of 2-year mortality, with optimal cutoff values around 45 [27]. Similarly, the Geriatric Nutritional Risk Index, though not achieving statistical significance in our multivariable models, has demonstrated consistent associations with adverse outcomes in elderly surgical populations [14, 28]. The attenuated significance of individual nutritional markers in our fully adjusted models likely reflects their integration into the composite CALLY and GINI indices, which may capture nutritional-inflammatory interactions more comprehensively than isolated parameters.

### Calibration and clinical utility

Beyond discrimination metrics, model calibration—the agreement between predicted probabilities and observed outcomes—is critical for clinical decision-making [41]. Our models demonstrated acceptable calibration across the observed probability range, as evidenced by Hosmer-Lemeshow goodness-of-fit statistics (χ² = 8.42–11.31, *p* > 0.05 for all models) and calibration plots showing close alignment between predicted and observed mortality rates across deciles of risk [23]. The Brier score, which quantifies the accuracy of probabilistic predictions (with lower values indicating better performance), ranged from 0.187 to 0.203 across our models—values consistent with well-calibrated prediction tools in geriatric surgery literature [42]. Notably, calibration-in-the-large (mean predicted risk: 28.2%; observed mortality: 28.8%) confirmed minimal systematic over- or under-prediction. The clinical utility of our integrated model extends beyond statistical metrics to practical risk stratification capacity. The clear separation of Kaplan-Meier survival curves by CALLY and GINI quartiles, with statistically significant differences emerging within 30 days post-surgery, enables early identification of high-risk patients during the critical perioperative window [43]. This early risk differentiation facilitates timely implementation of targeted interventions—including nutritional optimization, aggressive delirium prevention protocols, and intensified physiotherapy—that have demonstrated efficacy in reducing adverse outcomes in vulnerable elderly populations [32]. Furthermore, the reliance on routine preoperative laboratory values enhances the model’s pragmatic feasibility, requiring no additional testing beyond standard hip fracture workup and thus minimizing implementation barriers in resource-constrained settings [44].

The CALLY index, a novel composite biomarker integrating albumin, lymphocyte count, and CRP, has recently gained attention across diverse clinical contexts. In patients with acute myocardial infarction, CALLY values ≤ 3.03 predicted in-hospital mortality with 75.5% sensitivity and 78.2% specificity [16]. Among sepsis patients, CALLY demonstrated excellent discriminative ability (AUC 0.906) for 30-day mortality [18]. In elderly populations, higher CALLY values correlated with reduced all-cause and cardiovascular mortality [29]. Our study represents, to our knowledge, the first application of CALLY to hip fracture mortality prediction. While CALLY did not achieve independent statistical significance in multivariable regression (possibly due to collinearity with its constituent components), its robust risk stratification capability—with lowest quartile patients experiencing 40% 1-year mortality versus 18% in the highest quartile—demonstrates clear clinical utility. This stratification performance suggests that CALLY effectively synthesizes inflammatory, nutritional, and immunologic information into a single, readily calculable metric.

The GINI index, conceptualized as a measure of systemic inflammatory burden relative to nutritional-immune reserves, showed similar risk stratification capability in our cohort. Patients in the highest GINI quartile (greatest inflammation) experienced nearly 40% 1-year mortality compared to approximately 18% in the lowest quartile. While GINI has been less extensively studied than CALLY, its theoretical foundation—quantifying the imbalance between pro-inflammatory mediators and protective nutritional-immune factors—aligns with established pathophysiologic mechanisms underlying frailty and adverse surgical outcomes [30]. The early divergence of mortality curves within 30 days post-surgery suggests that GINI may capture acute perioperative inflammatory stress, complementing the more chronic vulnerability reflected by frailty scores.

The incremental improvements in risk reclassification observed with Models 2 and 3 (NRI 14.9% and 11.7%, respectively) merit consideration, as discussed in detail above regarding the trade-offs between model complexity and clinical utility. While traditional discrimination metrics (AUC) showed only modest gains, NRI quantifies the proportion of patients correctly reclassified into higher or lower risk categories—a clinically relevant measure when the goal is individualized risk stratification rather than binary prediction [31]. An NRI of approximately 20% for Model 2 indicates that one in five patients was more accurately classified by incorporating nutritional-inflammatory markers, potentially enabling more tailored perioperative management, such as targeted nutritional supplementation, enhanced monitoring, or modified surgical approaches for high-risk individuals [32].

Our study has several strengths. The multicentre design enhances generalizability, and the large sample size with complete 1-year follow-up minimizes attrition bias. The comprehensive assessment of frailty, nutritional, and inflammatory parameters, combined with rigorous statistical methodology including continuous NRI calculation and multiple timepoint analyses, provides robust evidence for the integrated model’s utility. The use of readily available laboratory values to calculate CALLY and GINI indices enhances clinical applicability, as these biomarkers require no additional testing beyond routine preoperative workup.

### Limitations

Several limitations warrant acknowledgment. First, the retrospective design precludes causal inference and may introduce unmeasured confounding. Second, while the models achieved acceptable discriminative performance (AUC 0.666–0.678), the modest sensitivity values (52.5%–56.4%) at standard probability thresholds indicate that a substantial proportion of patients who ultimately died were not classified as high-risk. This limitation, inherent to threshold-dependent metrics, can be partially addressed through threshold optimization or quartile-based stratification approaches, as discussed above [39]. Third, the observed increases in AIC and BIC across progressively complex models suggest diminishing returns in model parsimony, though this must be weighed against the clinically meaningful improvements in risk reclassification capacity [36]. Fourth, the paradoxical NHFS findings suggest potential model overfitting or multicollinearity, with variance inflation analysis revealing moderate collinearity between NHFS components and other frailty-nutritional variables (VIF 2.5–4.2). This statistical artifact underscores the challenges of incorporating multiple correlated predictors and suggests that future model refinements may benefit from dimension reduction or penalized regression techniques. Fifth, the modest discriminative performance (AUC 0.65–0.68) suggests that substantial mortality variance remains unexplained, likely attributable to unmeasured factors such as post-discharge care quality, social support, and rehabilitation intensity. Sixth, despite acceptable overall calibration (Hosmer-Lemeshow *p* > 0.05, Brier scores 0.187–0.203), calibration in extreme risk strata (very high or very low predicted probabilities) requires validation in larger datasets to ensure reliability across the full risk spectrum. Seventh, the lack of external validation limits generalizability, and the models require prospective validation in independent cohorts before clinical implementation. Eighth, the study population was drawn from tertiary care centers, potentially limiting applicability to community hospitals with different patient demographics and resource availability. Ninth, we did not assess inter-rater reliability for Clinical Frailty Scale assessments, which may introduce measurement variability given the instrument’s semi-subjective nature .Tenth, the absence of data on preoperative cognitive status and baseline activities of daily living—both established prognostic factors—represents an important gap. Finally, the GINI index, while theoretically sound, lacks extensive validation in geriatric surgical populations, and its optimal cutoff values remain to be established.

Future research should focus on external validation of the integrated CALLY-GINI-Frailty model in diverse healthcare settings and exploration of dynamic biomarker trajectories, as serial measurements may better capture evolving physiologic status than single preoperative assessments [45]. Investigation of targeted interventions—such as preoperative nutritional optimization or anti-inflammatory strategies—in high-risk patients identified by CALLY/GINI stratification could translate risk prediction into improved outcomes [46]. Additionally, incorporation of emerging biomarkers, such as inflammatory cytokines or microRNA profiles, may further refine prognostic accuracy [47].

## Conclusion

This multicentre cohort study demonstrates that integrating CALLY and GINI indices with clinical frailty assessments provides clinically meaningful risk stratification for mortality following hip fracture surgery. While discriminative performance remains modest (AUC 0.65–0.68) and severe multicollinearity limits coefficient interpretability, the models demonstrate excellent calibration and substantial risk reclassification improvements (NRI 14.9% for nutritional-inflammatory markers, additional 11.7% for CALLY/GINI). Threshold optimization to 0.30–0.35 is recommended to maximize sensitivity for screening purposes. The CALLY and GINI indices, derived from routine laboratory tests, offer practical tools for identifying high-risk patients who may benefit from intensified perioperative management and targeted interventions. Prospective external validation is essential to confirm these findings before widespread clinical implementation.

## Data Availability

No datasets were generated or analysed during the current study.

## References

[1] Veronese N, Maggi S (2018) Epidemiology and social costs of hip fracture. Injury 49(8):1458–1460. 10.1016/j.injury.2018.04.015 29699731 10.1016/j.injury.2018.04.015

[2] Forssten MP, Bass GA, Ismail AM, Mohseni S, Cao Y (2021) Predicting 1-year mortality after hip fracture surgery: an evaluation of multiple machine learning approaches. J Pers Med 11(8):727. 10.3390/jpm11080727 34442370 10.3390/jpm11080727 PMC8401745

[3] Sanz-Reig J, Mas-Martinez J, Ojeda-Thies C, Saez-Lopez MP, Alonso-García N, Gonzalez-Montalvo JI (2024) Emergency department prediction model for 30-day mortality after hip fracture: the Spanish National hip fracture registry (RNFC) cohort. Hip Int 34(2):290–297. 10.1177/11207000231197818 37670497 10.1177/11207000231197818

[4] Bernstein J, Lee A, Ahn J (2024) When does annual geriatric hip fracture mortality revert to baseline? Front Surg 11:1359648. 10.3389/fsurg.2024.1359648 39524966 10.3389/fsurg.2024.1359648 PMC11543560

[5] Karres J, Zwiers R, Eerenberg JP, Vrouenraets BC, Kerkhoffs GMMJ (2022) Mortality prediction in hip fracture patients: physician assessment versus prognostic models. J Orthop Trauma 36(11):585–592. 10.1097/BOT.0000000000002412 35605101 10.1097/BOT.0000000000002412 PMC9555757

[6] Walsh ME, Kristensen PK, Hjelholt TJ et al (2024) Systematic review of multivariable prognostic models for outcomes at least 30 days after hip fracture finds 18 mortality models but no nonmortality models warranting validation. J Clin Epidemiol 173:111439. 10.1016/j.jclinepi.2024.111439 38925343 10.1016/j.jclinepi.2024.111439

[7] Nordström P, Ahlqvist VH, Ballin M, Nordström A (2024) A novel clinical prediction model for hip fractures: a development and validation study in the total population of Sweden. EClinicalMedicine 77:102877. 10.1016/j.eclinm.2024.102877 39430614 10.1016/j.eclinm.2024.102877 PMC11490797

[8] Patel KV, Brennan KL, Brennan ML, Jupiter DC, Shar A, Davis ML (2014) Association of a modified frailty index with mortality after femoral neck fracture in patients aged 60 years and older. Clin Orthop Relat Res 472(3):1010–1017. 10.1007/s11999-013-3334-7 24166073 10.1007/s11999-013-3334-7 PMC3916591

[9] Dent E, Martin FC, Bergman H, Woo J, Romero-Ortuno R, Walston JD (2019) Management of frailty: opportunities, challenges, and future directions. Lancet 394(10206):1376–1386. 10.1016/S0140-6736(19)31785-4 31609229 10.1016/S0140-6736(19)31785-4

[10] Yan B, Sun W, Wang W, Wu J, Wang G, Dou Q (2022) Prognostic significance of frailty in older patients with hip fracture: a systematic review and meta-analysis. Int Orthop 46(12):2939–2952. 10.1007/s00264-022-05605-9 36227383 10.1007/s00264-022-05605-9

[11] Song Y, Wu Z, Huo H, Zhao P (2022) The impact of frailty on adverse outcomes in geriatric hip fracture patients: a systematic review and meta-analysis. Front Public Health 10:890652. 10.3389/fpubh.2022.890652 35844855 10.3389/fpubh.2022.890652 PMC9280195

[12] Malafarina V, Reginster JY, Cabrerizo S et al (2018) Nutritional status and nutritional treatment are related to outcomes and mortality in older adults with hip fracture. Nutrients 10(5):555. 10.3390/nu10050555 29710860 10.3390/nu10050555 PMC5986435

[13] Popp D, Stich-Regner M, Schmoelz L, Silvaieh S, Heisinger S, Nia A (2024) Predictive feasibility of the Graz malnutrition Screening, controlling nutritional status Score, geriatric nutritional risk index, and prognostic nutritional index for postoperative long-term mortality after surgically treated proximal femur fracture. Nutrients 16(24):4280. 10.3390/nu16244280 39770903 10.3390/nu16244280 PMC11676286

[14] Mazzola P, Bellelli G, Broggini V et al (2015) Postoperative delirium and pre-fracture disability predict 6-month mortality among the oldest old hip fracture patients. Aging Clin Exp Res 27(1):53–60. 10.1007/s40520-014-0242-y 24880696 10.1007/s40520-014-0242-y

[15] Özdemir E, Özdeş OO, Topsakal FE, Altay N, Demirel E (2025) The impact of frailty indices on predicting complications and functional recovery in proximal humerus fractures: a comparative study. Med (Kaunas) 61(7):1169. 10.3390/medicina61071169 10.3390/medicina61071169 PMC12298698 40731798

[16] Demir Y, Sevinc S (2025) The prognostic value of C-reactive protein–albumin–lymphocyte (CALLY) index in predicting in-hospital mortality after primary percutaneous coronary intervention in patients with ST-elevation myocardial infarction (STEMI). Catheter Cardiovasc Interv 106(2):1111–1118. 10.1002/ccd.31623 40452651 10.1002/ccd.31623

[17] Wen Y, Zhou Z, Ou Y, Ye P, Tang Y, Zou Q (2025) Prognostic significance of the CALLY index for cancer risk and survival: evidence from NHANES 2001–2018. World J Surg Oncol 23(1):431. 10.1186/s12957-025-04094-5 41219785 10.1186/s12957-025-04094-5 PMC12607156

[18] Yılmaz E, Ak R (2025) Evaluation of the C-reactive protein–albumin–lymphocyte (CALLY) index as a prognostic marker in patients with sepsis. BMC Emerg Med 25(1):194. 10.1186/s12873-025-01356-z 41023805 10.1186/s12873-025-01356-z PMC12482414

[19] Bouillanne O, Morineau G, Dupont C et al (2005) Geriatric nutritional risk index: a new index for evaluating at-risk elderly medical patients. Am J Clin Nutr 82(4):777–783. 10.1093/ajcn/82.4.777 16210706 10.1093/ajcn/82.4.777

[20] Kaizu Y, Tamura S, Saito H, Hayashi S, Iwamoto H, Miyata K (2023) Clinical prediction models for nonmortality outcomes in older adults with hip fractures: A systematic review. J Gerontol Biol Sci Med Sci 78(12):2363–2370. 10.1093/gerona/glad205 10.1093/gerona/glad205 37607009

[21] Morri M, Ambrosi E, Chiari P et al (2019) One-year mortality after hip fracture surgery and prognostic factors: a prospective cohort study. Sci Rep 9(1):18718. 10.1038/s41598-019-55196-6 31822743 10.1038/s41598-019-55196-6 PMC6904473

[22] Heyes GJ, Tucker A, Marley D, Foster A (2017) Predictors for 1-year mortality following hip fracture: a retrospective review of 465 consecutive patients. Eur J Trauma Emerg Surg 43(1):113–119. 10.1007/s00068-015-0556-2 26260068 10.1007/s00068-015-0556-2

[23] Hosmer DW, Lemeshow S, Sturdivant RX (2013) Applied logistic regression, 3rd edn. Wiley, Hoboken, NJ. 10.1002/9781118548387

[24] Kristensen PK, Thillemann TM, Søballe K, Johnsen SP (2016) Can improved quality of care explain the success of orthogeriatric units? A population-based cohort study. Age Ageing 45(1):66–71. 10.1093/ageing/afv155 26582757 10.1093/ageing/afv155

[25] Moppett IK, Parker M, Griffiths R, Bowers T, White SM, Moran CG (2012) Nottingham hip fracture score: longitudinal and multi-assessment. Br J Anaesth 109(4):546–550. 10.1093/bja/aes187 22728204 10.1093/bja/aes187

[26] Xu Y, Luo Y (2025) Preoperative prognostic nutritional index is a predictive factor for postoperative delirium in elderly patients with femoral neck fracture. Clin Interv Aging 20:941–950. 10.2147/CIA.S518366 40621090 10.2147/CIA.S518366 PMC12229154

[27] Helminen H, Luukkaala T, Saarnio J, Nuotio M (2017) Comparison of the Mini-Nutritional assessment short and long form and serum albumin as prognostic indicators of hip fracture outcomes. Injury 48(4):903–908. 10.1016/j.injury.2017.02.007 28249678 10.1016/j.injury.2017.02.007

[28] Kang MK, Kim TJ, Kim Y et al (2020) Geriatric nutritional risk index predicts poor outcomes in patients with acute ischemic stroke: automated undernutrition screen tool. PLoS ONE 15(2):e0228738. 10.1371/journal.pone.0228738 32053672 10.1371/journal.pone.0228738 PMC7017988

[29] GBofCDandR2023C (2025) Global, regional, and National burden of cardiovascular diseases and risk factors in 204 countries and territories, 1990–2023. J Am Coll Cardiol 86(22):2167–2243. 10.1016/j.jacc.2025.08.015 40990886 10.1016/j.jacc.2025.08.015

[30] Ferrucci L, Fabbri E (2018) Inflammageing: chronic inflammation in ageing, cardiovascular disease, and frailty. Nat Rev Cardiol 15(9):505–522. 10.1038/s41569-018-0064-2 30065258 10.1038/s41569-018-0064-2 PMC6146930

[31] Pencina MJ, D’Agostino RB, Sr, Steyerberg EW (2011) Extensions of net reclassification improvement calculations to measure usefulness of new biomarkers. Stat Med 30(1):11–21. 10.1002/sim.4085 21204120 10.1002/sim.4085 PMC3341973

[32] Weimann A, Braga M, Carli F et al (2017) ESPEN guideline: clinical nutrition in surgery. Clin Nutr 36(3):623–650. 10.1016/j.clnu.2017.02.013 28385477 10.1016/j.clnu.2017.02.013

[33] Leeflang MM, Rutjes AW, Reitsma JB, Hooft L, Bossuyt PM (2013) Variation of a test’s sensitivity and specificity with disease prevalence. CMAJ 185(11):E537–E544. 10.1503/cmaj.121286 23798453 10.1503/cmaj.121286 PMC3735771

[34] Moons KG, Altman DG, Reitsma JB et al (2015) Transparent reporting of a multivariable prediction model for individual prognosis or diagnosis (TRIPOD): explanation and elaboration. Ann Intern Med 162(1):W1–W73. 10.7326/M14-0698 25560730 10.7326/M14-0698

[35] YOUDEN WJ (1950) Index for rating diagnostic tests. Cancer 3(1):32–35. 10.1002/1097-0142(1950)3:1%3C32::aid-cncr2820030106%3E3.0.co;23 15405679

[36] Vickers AJ, Elkin EB (2006) Decision curve analysis: a novel method for evaluating prediction models. Med Decis Mak 26(6):565–574. 10.1177/0272989X06295361 10.1177/0272989X06295361 PMC2577036 17099194

[37] Van Calster B, Wynants L, Verbeek JFM et al (2018) Reporting and interpreting decision curve analysis: A guide for investigators. Eur Urol 74(6):796–804. 10.1016/j.eururo.2018.08.038 30241973 10.1016/j.eururo.2018.08.038 PMC6261531

[38] Collins GS, Reitsma JB, Altman DG, Moons KG (2015) Transparent reporting of a multivariable prediction model for individual prognosis or diagnosis (TRIPOD): the TRIPOD statement. BMJ 350:g7594. 10.1136/bmj.g7594 25569120 10.1136/bmj.g7594

[39] Thompson CG, Kim RS, Aloe AM, Becker BJ (2017) Extracting the variance inflation factor and other multicollinearity diagnostics from typical regression results. Basic Appl Soc Psych 39(2):81–90. 10.1080/01973533.2016.1277529

[40] Tu YK, Clerehugh V, Gilthorpe MS (2004) Collinearity in linear regression is a serious problem in oral health research. Eur J Oral Sci 112(5):389–397. 10.1111/j.1600-0722.2004.00160.x 15458496 10.1111/j.1600-0722.2004.00160.x

[41] Alba AC, Agoritsas T, Walsh M, Hanna S, Iorio A, Devereaux PJ, McGinn T, Guyatt G (2017) Discrimination and calibration of clinical prediction models: users’ guides to the medical literature. JAMA 318(14):1377–1384. 10.1001/jama.2017.12126 29049590 10.1001/jama.2017.12126

[42] Steyerberg EW, Vickers AJ (2008) Decision curve analysis: a discussion. Med Decis Mak 28(1):146–149. 10.1177/0272989X07312725 10.1177/0272989X07312725 PMC2577563 18263565

[43] Moja L, Piatti A, Pecoraro V et al (2012) Timing matters in hip fracture surgery: patients operated within 48 hours have better outcomes. A meta-analysis and meta-regression of over 190,000 patients. PLoS ONE 7(10):e46175. 10.1371/journal.pone.0046175 23056256 10.1371/journal.pone.0046175 PMC3463569

[44] Moons KGM, Wolff RF, Riley RD et al (2019) PROBAST: A tool to assess risk of bias and applicability of prediction model studies: explanation and elaboration. Ann Intern Med 170(1):W1–W33. 10.7326/M18-1377 30596876 10.7326/M18-1377

[45] Buta BJ, Walston JD, Godino JG et al (2016) Frailty assessment instruments: systematic characterization of the uses and contexts of highly-cited instruments. Ageing Res Rev 26:53–61. 10.1016/j.arr.2015.12.003 26674984 10.1016/j.arr.2015.12.003 PMC4806795

[46] Deutz NE, Matheson EM, Matarese LE, NOURISH Study Group et al (2016) Readmission and mortality in malnourished, older, hospitalized adults treated with a specialized oral nutritional supplement: a randomized clinical trial. Clin Nutr 35(1):18–26. 10.1016/j.clnu.2015.12.010 26797412 10.1016/j.clnu.2015.12.010

[47] Bellavia D, Dimarco E, Costa V et al (2021) Flavonoids in bone erosive diseases: perspectives in osteoporosis treatment. Trends Endocrinol Metab 32(2):76–94. 10.1016/j.tem.2020.11.007 33288387 10.1016/j.tem.2020.11.007

